# Estratégias adotadas na América do Sul para a gestão da infodemia da COVID-19

**DOI:** 10.26633/RPSP.2021.43

**Published:** 2021-05-12

**Authors:** Cristianne Aparecida Costa Haraki

**Affiliations:** 1 Ministério da Saúde, Secretaria de Vigilância Sanitária Brasília (DF) Brasil Ministério da Saúde, Secretaria de Vigilância Sanitária, Brasília (DF), Brasil.

**Keywords:** Infecções por Coronavirus, pandemias, acesso à informação, atenção à saúde, Coronavirus infections, pandemics, access to information, health care (public health), Infecciones por coronavírus, pandemias, acceso a la información, atención a la salud

## Abstract

**Objetivo.:**

Identificar a existência ou ausência de estratégias para a gestão de infodemia nos ministérios da saúde de 10 países da região da América do Sul (Brasil, Argentina, Uruguai, Paraguai, Bolívia, Peru, Chile, Colômbia, Equador, Venezuela).

**Métodos.:**

Definiu-se “estratégia” como o uso planejado de recursos para atingir ou concretizar determinados objetivos — sendo, neste caso, considerado como objetivo o combate à infodemia. Os *sites* eletrônicos oficiais dos ministérios da saúde dos países foram examinados de 28 de setembro a 10 de outubro de 2020 em busca de materiais, páginas eletrônicas ou aplicativos que sugerissem a existência de uma estratégia de combate à infodemia; informações sobre infodemia para cidadãos; e materiais comunicacionais como vídeos e cartazes. Ainda, utilizando “infodemia” como palavra-chave, foram buscados documentos oficiais (normativas, decretos, protocolos) que mencionassem medidas relacionadas à gestão da infodemia. Os resultados foram classificados em três categorias: existência de estratégia de gestão; existência de alguma ação de combate à infodemia; e ausência de informação sobre infodemia.

**Resultados.:**

Dos 10 países analisados, apenas a Argentina possuía uma estratégia destinada à gestão da infodemia. Brasil, Chile, Equador e Paraguai apenas faziam menção ao tema no *site* de seus respectivos ministérios da saúde; e nenhuma menção foi encontrada nos *sites* dos ministérios da saúde de Bolívia, Colômbia, Peru, Uruguai e Venezuela.

**Conclusões.:**

Os países estudados não fazem uso do conhecimento disponível acerca de estratégias de combate e gestão da infodemia.

## Abstract (en)

**Objective.:**

To identify evidence of infodemic management strategies in the ministry of health websites in 10 South American countries (Brazil, Argentina, Uruguay, Paraguay, Bolivia, Peru, Chile, Colombia, Ecuador, Venezuela).

**Method.:**

“Strategy” was defined as the planned use of resources to achieve of materialize certain objectives — in this case, fighting the infodemic. The official ministry of health websites in each country were examined from 28 September to 10 October 2020 in search of materials, electronic pages or apps suggesting the existence of an infodemic management strategy, information about the infodemic for citizens, and communication materials such as videos and posters. Additionally, using “infodemic” as a keyword, official documents (rulings, decrees, protocols) mentioning infodemic management measures were searched. The results were classified into three categories: existence of an infodemic management strategy; existence of infodemic control actions; absence of information on the topic.

**Results.:**

Of the 10 countries analyzed, only Argentina had an infodemic management strategy. Infodemic was mentioned as a topic in the ministry of health websites in Brazil, Chile, Ecuador, and Paraguay; and no mention was identified in the ministry of health websites in Bolivia, Colombia, Peru, Uruguay, and Venezuela.

**Conclusions.:**

The studied countries do not make use of the available knowledge regarding infodemic management strategies.

## RESUMEN

**Objetivo.:**

Determinar la existencia o la falta de estrategias para la gestión de la infodemia en los Ministerios de Salud de diez países de América del Sur (Argentina, Bolivia, Brasil, Colombia, Chile, Ecuador, Paraguay, Perú, Uruguay y Venezuela).

**Métodos.:**

Se definió el término “estrategia” como el uso planificado de recursos para alcanzar o concretar determinados objetivos y, para este último fin, se fijó como objetivo la lucha contra la infodemia. Del 28 de septiembre al 10 de octubre del 2020, se examinaron los sitios electrónicos oficiales de los Ministerios de Salud de los países en busca de materiales, páginas electrónicas o aplicaciones que indicaran la existencia de una estrategia para combatir la infodemia, datos sobre la infodemia para la ciudadanía y materiales de comunicación como videos y carteles. Con el término “infodemia” como palabra clave, se buscaron documentos oficiales (directrices, decretos y protocolos) que mencionaran medidas relacionadas con la gestión de la infodemia. Los resultados se clasificaron en tres categorías: la existencia de una estrategia de gestión, la existencia de alguna medida para combatir la infodemia y la falta de datos sobre la infodemia.

**Resultados.:**

Entre los diez países analizados, solamente Argentina tenía una estrategia destinada a la gestión de la infodemia. Brasil, Chile, Ecuador y Paraguay apenas mencionaban el tema en el sitio web de sus respectivos Ministerios de Salud; y no se encontró ninguna mención en los sitios web de los Ministerios de Salud de Bolivia, Colombia, Perú, Uruguay y Venezuela.

**Conclusiones.:**

Los países estudiados no utilizan el conocimiento disponible acerca de las estrategias para combatir y gestionar la infodemia.

A infodemiologia é definida como a “ciência da distribuição e dos determinantes da informação em meio eletrônico, especificamente a Internet, ou na população, com o objetivo final de informar sobre saúde pública e política pública” (1, p.3). O termo foi utilizado pela primeira vez na literatura científica em 2002 por Gunther Eysenbach (1, 2). No entanto, estudos que abordavam a infodemiologia como conceito são conhecidos desde 1996 (1). Por sua vez, o fenômeno da infodemia ocorre quando essa distribuição de informação se torna excessiva e, muitas vezes, pouco confiável, sem fontes claras e contendo orientações pouco fidedignas (3).

Em contextos de epidemia, a disseminação de rumores, teorias da conspiração e informações que causam estigma em determinado grupo populacional ou profissional tem sido observada na literatura desde a gripe espanhola até tempos mais recentes — por exemplo, nas epidemias de HIV, Zika vírus e Ebola (4-6). No caso da gripe espanhola, em 1918 os jornais brasileiros começaram a publicar notas sobre uma epidemia que atacava a Europa. O aumento do volume de publicações foi acompanhado pelo crescimento do medo por parte da população, havendo relatos, inclusive, de suicídios causados pelos temores que as notícias veiculadas causavam (4). Em todas essas epidemias, informações falsas ou enganosas interferiram na preparação e resposta à emergência em saúde pública causada pela doença.

Em relação ao novo coronavírus, desde o relato, em dezembro de 2019, de uma pneumonia de causa desconhecida na província de Wuhan, China, até os primeiros casos registrados fora da China, na Tailândia e nos Estados Unidos (7) e a declaração de emergência de saúde pública de importância internacional (ESPII) pela Organização Mundial da Saúde (OMS) (8), a pandemia da COVID-19 tem sido notadamente influenciada por uma infodemia. O diretor-geral da OMS, Tedros Adhanom Ghebreyesus, chegou a afirmar, na Conferência de Segurança de Munique 2020, que “nós não estamos apenas lutando contra a pandemia, nós estamos lutando contra uma infodemia” (9). A Organização para a Cooperação e Desenvolvimento Econômico (OCDE), em um documento que orienta os governos a combaterem a desinformação sobre COVID-19 nas plataformas digitais, aprofundou o tema, abordando conceitos como má informação (disseminação de informação falsa independentemente da intenção de enganar) e desinformação (disseminação deliberada de informação falsa ou enganosa com a intenção de enganar) e suas respectivas consequências (10).

As características da sociedade globalizada em que vivemos, que afetam e facilitam a maneira como nos relacionamos, nos comunicamos, nos locomovemos e nos transportamos, foram fatores cruciais para o espalhamento do vírus em nível global e para o estabelecimento do estado de pandemia do novo coronavírus. Assim como o vírus, as informações sobre ele também se espalharam com rapidez. A Internet, por meio das páginas das grandes agências de notícias, plataformas de busca, redes sociais e plataformas de compartilhamento de vídeos, assim como dos aplicativos de troca de mensagens, tem sido o canal de propagação da infodemia, disseminando tanto informações de utilidade para o combate à pandemia da COVID-19 como informações tóxicas.

Um estudo feito em seis países (Argentina, Alemanha, Coreia do Sul, Espanha, Estados Unidos e Reino Unido) (11), em março e abril de 2020, buscou compreender como as pessoas buscam notícias e informações sobre a COVID-19, como elas classificam a confiabilidade das diferentes fontes e plataformas buscadas, que grau de desinformação elas encontraram e o conhecimento e resposta dos entrevistados sobre a crise do coronavírus. O estudo demonstrou o aumento do uso e da busca de notícias e informações em *sites* de busca, aplicativos de troca de mensagens, redes sociais e plataformas de vídeo, às vezes combinando esses canais. Nos seis países analisados, observou-se que entrevistados com menor grau de educação formal tinham mais tendência a confiar em informações de redes sociais e aplicativos de mensagens do que em empresas de notícias. Por outro lado, em cada país analisado, um alto número de entrevistados — de diversas faixas etárias, níveis de educação e preferência política — classificaram cientistas, doutores e outros especialistas em saúde como fontes confiáveis de informação sobre a COVID-19. A maioria dos entrevistados classificou as plataformas digitais como menos confiáveis que os especialistas, autoridades de saúde e agências de notícias.

Outro estudo (5) coletou relatos relacionados à infodemia em diversos canais da Internet de 31 de dezembro de 2019 a 5 de abril de 2020. Foram identificados 2 311 relatos relacionados à infodemia da COVID-19, em 25 línguas e 87 países. Desses, 89% foram classificados como rumores, 7,8% como teoria da conspiração e 3,5% como estigmatizantes. Do total de relatos passíveis de classificação, 82% foram considerados falsos, 9% continham informações corretas, 8% continham informações enganosas e 1% continha informações que não puderam ser comprovadas. A origem da maior parte desses materiais foi a Índia, Estados Unidos, China, Espanha, Indonésia e Brasil.

Diante da relevância do tema, o presente estudo teve como objetivo identificar a existência de estratégias para a gestão de infodemia em países selecionados da região da América do Sul no contexto do combate à pandemia da COVID-19.

## MATERIAIS E MÉTODOS

Para a realização deste estudo, foram selecionados países da América do Sul que adotam como língua oficial o espanhol e o português: Argentina, Bolívia, Brasil, Chile, Colômbia, Equador, Paraguai, Peru, Uruguai e Venezuela. Foram excluídos, portanto, os países que não se enquadraram nesse critério: Guiana, Guiana Francesa e Suriname. Uma vez definida a amostra, definiu-se como como fonte de informações a autoridade máxima na gestão da saúde em cada país, ou seja, os ministérios da saúde.

Para determinar se e como os países estão realizando a gestão da infodemia, foram realizadas análises detalhadas dos *sites* oficiais dos ministérios da saúde de todos os países. Os conteúdos e materiais disponibilizados nos *sites* foram examinados em busca de evidências de uma estratégia estruturada de combate à infodemia. Foram buscados conteúdos disponibilizados para cidadãos em formato de “informação” ou “comunicação” que se referissem à infodemia e analisados os materiais comunicacionais disponíveis para uso público, como vídeos e cartazes. Finalmente, buscaram-se normativas, decretos, protocolos e outros documentos oficiais que mencionassem medidas relacionadas à gestão da infodemia e que estivessem disponíveis nos referidos *sites*. O termo “infodemia” foi utilizado como palavra-chave principal na busca por estratégias, ações, materiais e normativas.

Como o objetivo deste estudo foi identificar a existência de estratégias, foi preciso definir o que se considera como estratégia para a seleção dos achados. Assim, consideraram-se os seguintes conceitos para estratégia: “arte de utilizar planejadamente os recursos de que se dispõe ou de explorar de maneira vantajosa a situação ou as condições favoráveis de que porventura se desfrute, de modo a atingir determinados objetivos” (12); e “caminhos escolhidos que indicam como a organização pretende concretizar seus objetivos e, consequentemente sua missão” (13, p. 39). Foram consideradas as estratégias que atendiam a definição e que tivessem como foco o combate à infodemia.

Os resultados foram classificados como: existência de estratégia de gestão; existência de alguma ação isolada de combate à infodemia; e ausência de informação sobre infodemia. As buscas foram realizadas de 28 de setembro a 10 de outubro de 2020.

## RESULTADOS

Os resultados refletem as informações encontradas nos *sites* eletrônicos dos ministérios da saúde dos 10 países estudados e que estavam disponíveis para consulta pública no período da pesquisa. Dos 10 países analisados, apenas a Argentina possuía uma estratégia destinada à gestão da infodemia no momento da pesquisa. Brasil, Chile, Equador e Paraguai apenas faziam menção ao tema no *site* de seus respectivos ministérios da saúde; e nenhuma menção foi encontrada nos *sites* dos ministérios da saúde de Bolívia, Colômbia, Peru, Uruguai e Venezuela. No caso do Peru, foi encontrado na literatura um artigo que menciona um decreto do Ministério da Justiça que determina pena de detenção para disseminadores de *fake news* no país (14).

### Argentina

O governo argentino criou a plataforma “Confiar – Infodemia, a epidemia informativa da pandemia” (https://confiar.telam.com.ar/). A Plataforma Confiar é uma iniciativa da Secretaria de Meios e Comunicação Pública do governo argentino, desenvolvida por meio da Agência Nacional de Notícias Télam.

Essa plataforma oferece três serviços destinados ao cidadão. O primeiro é o “Verdadeiro ou falso”, que reúne tudo o que é preciso saber sobre a COVID-19 em uma página interativa contendo várias afirmações sobre as quais o cidadão clica, recebendo confirmação sobre a afirmação ser verdadeira ou falsa por meio de um texto explicativo. O segundo serviço é o “*Fake News”,* que apresenta as notícias falsas sobre o coronavírus que circulam na Argentina, explicando cada uma delas. Os conteúdos desmentidos incluem: vacinas contra a COVID-19 são produzidas com células de fetos abortados; as vacinas causam esterilidade em homens; as vacinas contêm um tipo de mercúrio que pode causar danos neurológicos. Por fim, a seção “Caixa de ferramentas” oferece estratégias para detectar a informação falsa. Também é uma página interativa, com orientações para proteção contra a infodemia, apelo para que o cidadão reflita sobre as notícias e sobre como se sente em relação a elas e canal para denúncia de notícias e perfis de redes sociais.

A plataforma traz, ainda, vídeos de especialistas esclarecendo a população sobre alguns temas, notícias da Argentina, do mundo e da OMS e os números da pandemia no país e no mundo. Apesar da relevância da estratégia no atual momento, em que a infodemia é uma ameaça constante aos esforços dos países em combater a pandemia da COVID-19, o acesso à plataforma Confiar, pelo *site* do Ministério da Saúde argentino, não é simples e direto. Para chegar à plataforma é preciso acessar a página de informações sobre a COVID-19 (https://www.argentina.gob.ar/salud/coronavirus-COVID-19), depois localizar a seção de “perguntas frequentes” e, finalmente, acessar a pergunta “Onde posso consultar informação atualizada e verídica sobre COVID-19”.

### Brasil

O *site* do Ministério da Saúde do Brasil traz uma iniciativa de combate às notícias falsas que pode ser acessada por meio da plataforma “Coronavírus COVID-19” (https://coronavirus.saude.gov.br/). A partir daí, é possível acessar a seção denominada “*Fake News”* que, por sua vez, direciona o cidadão para informação sobre as ações do Ministério para combater esse fenômeno. O Ministério disponibilizou um número exclusivo no aplicativo WhatsApp para que a população possa enviar informações recebidas nas redes sociais, que são então apuradas pelas áreas técnicas e respondidas oficialmente com relação à veracidade ou não da informação enviada. Esse canal, denominado “Saúde sem *fake news*”, não é exclusivo para a COVID-19, mas oferece uma seção exclusiva que trata de notícias falsas sobre o coronavírus.

### Chile

O Ministério da Saúde do Chile disponibiliza em seu *site* (https://www.minsal.cl/) uma função chamada “Mitos COVID-19” que, ao ser acessada, direciona o cidadão para o *site* da OMS com “conselhos para a população acerca dos rumores sobre o novo coronavírus (2019-nCoV)” (https://www.who.int/es/emergencies/diseases/novel-coronavirus-2019/advice-for-public/myth-busters).

### Equador

O Ministério da Saúde do Equador disponibiliza em seu *site* um canal pelo qual o cidadão pode obter informações sobre medidas de proteção (https://www.salud.gob.ec/medidas-de-proteccion-basicas-contra-el-nuevo-coronavirus/). Dentre as medidas apresentadas, há um item (*Canales oficiales*) que fornece a seguinte informação: “Informe-se por canais oficiais. Evite compartilhar rumores ou notícias que não tenham sido confirmadas. Solicitamos respeito à família dos pacientes e àqueles que fazem parte do grupo de contato”.

### Paraguai

Não foi encontrada nenhuma seção dedicada à infodemia no *site* do Ministério da Saúde paraguaio (https://www.mspbs.gov.py/index.php). O canal específico sobre COVID-19 oferece uma seção contendo informações para cidadãos e de comunicação (https://www.mspbs.gov.py/comunicacion-covid19.html) que apresenta dois vídeos curtos, um dos quais alerta para a confusão que o grande volume de informações pode gerar e para a necessidade de utilizar fontes seguras de informação; o segundo vídeo (*Información veraz*) é um comunicado do ministro da saúde que ressalta que o governo oferece informações seguras e dados atualizados, porém sem alusão à infodemia. Um comunicado divulgado no início da pandemia no país, em março de 2020, alerta sobre notícias falsas de novos casos de COVID-19 divulgadas nas redes sociais e reitera a necessidade da comprovação das fontes das notícias (https://www.mspbs.gov.py/dependencias/portal/fotos/ac3c38-Informacionesfalsas12.03.20.jpg)

## DISCUSSÃO

As consequências da infodemia para a saúde pública são graves, porque induzem a mudança de comportamento e envolvem a exposição das pessoas a fatores de risco (como a recusa em usar máscaras na pandemia da COVID-19), tratamentos, medicamentos e substâncias que podem levar a desfechos fatais. Ademais, os sistemas e profissionais de saúde também são vítimas da infodemia quando há geração de pânico na população e corrida para comprar equipamentos e medicamentos que podem fazer falta onde de fato são necessários. Além da saúde pública, a profusão de informações falsas e enganosas fomenta a discriminação e serve para que empresas e pessoas inidôneas obtenham proveitos financeiros a partir de uma situação de grave crise sanitária e humanitária. Como consequência final, há atraso e prejuízo na implementação de ações de preparação e resposta exitosas à pandemia da COVID-19.

Apesar da grande quantidade e qualidade dos conhecimentos já gerados sobre as consequências negativas que a infodemia pode causar para a saúde pública de um país, uma região e do mundo, os resultados encontrados sugerem que esse tema ainda não está sendo abordado com a importância, metodologias e urgência requeridas nos países estudados. Mesmo considerando que o estudo analisou as informações e materiais disponíveis nos *sites* apenas dos ministérios da saúde, os resultados encontrados foram muito tímidos, com apenas um dos 10 países analisados tratando o tema da infodemia de maneira clara na sua agenda de combate à pandemia da COVID-19.

Vale notar ainda que, em todos os países onde alguma informação direta ou indiretamente relacionada à infodemia foi identificada, o acesso aos conteúdos era difícil e pouco intuitivo. A maior parte das informações estava disponível apenas de maneira secundária nos *sites* dos ministérios da saúde. Assim, para encontrar essas informações, o cidadão precisaria de conhecimento prévio acerca do caminho a ser seguido até o conteúdo, ou dependeria de algum grau de alfabetização digital para ser capaz de explorar os *sites*.

**FIGURA 1.**
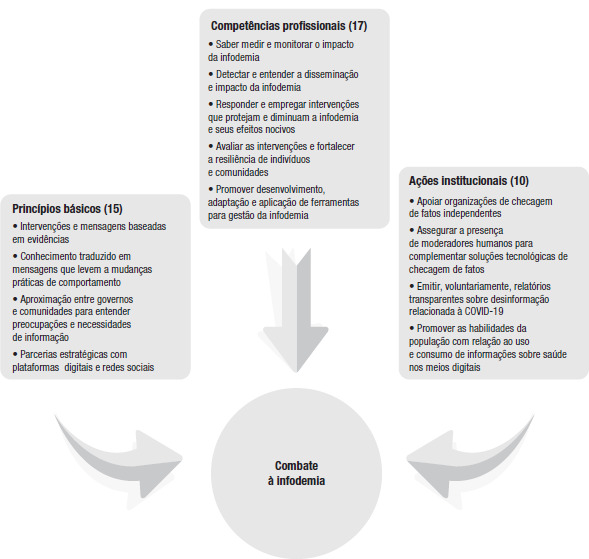
Estratégias de combate à infodemia sugeridas por organizações internacionais

Eysenbach, o pioneiro no uso do termo “infodemiologia”, propõe uma estrutura com quatro pilares para gestão da infodemia: 1) informação e monitoramento (infovigilância); 2) promoção da alfabetização em saúde digital e científica; 3) promoção do aperfeiçoamento do conhecimento e processo de melhora da qualidade da informação com checagem de fatos e revisão por pares; e 4) tradução acurada e tempestiva do conhecimento, minimizando fatores de distorção tais como fatores políticos ou influência comercial (2). Esses fatores são contemplados em princípios propostos pela OMS (15) e ações propostas pela OCDE (10) para governos e plataformas para combater a infodemia (figura 1).

De fato, a OMS, como principal líder do combate global à pandemia da COVID-19, lidera a discussão sobre gestão da infodemia, definida como o “uso de intervenções baseadas em evidências que trazem informações localizadas baseadas em evidência, compreensíveis para os cidadãos, e levam a um comportamento positivo de busca por saúde” (15). Em abril de 2020, a OMS realizou uma consulta pública para a elaboração de uma estrutura de gestão da infodemia. Em junho do mesmo ano, a OMS realizou a Primeira Conferência sobre Infodemiologia (15); e de outubro a dezembro, realizou a Terceira Conferência Virtual Global de Gestão da Infodemia (16). Ademais, a OMS ofertou, em novembro de 2020, o primeiro treinamento em gestão da infodemia para 275 profissionais selecionados, quando foram apresentadas as competências necessárias aos profissionais para a gestão da infodemia (17).

Como se observa pelo exposto, existem diversos atores propondo estratégias para a gestão da infodemia, que podem e devem ser aproveitadas pelos países da região da América do Sul para mitigar os efeitos negativos no contexto da pandemia da COVID-19. Porém, foram escassos os princípios, estruturas e orientações encontrados nos cinco países onde havia alguma estratégia ou ação voltada para o combate da infodemia. A Argentina elaborou uma plataforma específica para tratar do tema e o fez por meio da sua Agência Nacional de Notícias e não pelo seu Ministério da Saúde. Além disso, oferece uma ferramenta para que o cidadão se capacite na identificação e denúncia de notícias falsas. Por sua vez, Brasil, Chile, Equador e Paraguai ressaltaram, nos *sites* de seus ministérios da saúde, a importância das informações baseadas em evidências e mudança de comportamento, mas, em sua maioria, replicaram informações de outras fontes.

Em conclusão, várias propostas para gestão da infodemia já existem — ou seja, os países têm à sua disposição apoio de atores importantes no cenário da saúde global e informações vindas de autoridades internacionais de saúde pública e do meio científico que podem auxiliá-los a desenvolver estratégias mais competentes de combate à infodemia. Cabe então perguntar se os países estão considerando essas possibilidades de apoio e uso de informação adequada para a construção de suas estratégias de gestão da infodemia.

Apesar das limitações do presente estudo, que coletou informações apenas nos *sites* dos ministérios da saúde dos países estudados, sabe-se que a pandemia da COVID-19 afetou vários setores de governo além do setor saúde. Assim como a estratégia da Argentina está estruturada no setor de comunicação, o Peru tomou alguma iniciativa no campo da justiça (14). Seria, portanto, necessário ampliar o estudo para outras áreas de governo e áreas não governamentais como redes sociais, aplicativos de trocas de mensagens e grandes agências de notícias. Entretanto, o presente trabalho pôde mostrar que nos países estudados, do ponto de vista dos ministérios da saúde — protagonistas no combate à pandemia da COVID-19 —, ainda há muito a ser construído para uma gestão bem-sucedida da infodemia.

## Declaração.

As opiniões expressas no manuscrito são de responsabilidade exclusiva dos autores e não refletem necessariamente a opinião ou política da RPSP/PAJPH ou da Organização Pan-Americana da Saúde (OPAS).
